# VESTA: A GROUNDBREAKING TOOL FOR ENHANCING FINANCIAL LITERACY AND SCAM RESILIENCE AMONG OLDER ADULTS

**DOI:** 10.1093/geroni/igae098.3559

**Published:** 2024-12-31

**Authors:** Michael Barnett, Samuel Van Vleet, Rebekah Griffin, Matthew Fontanese, William Mallender, Harrison Boynton, Allyson Coldiron

**Affiliations:** The University of Texas Tyler, Tyler, Texas, United States; Miami University, Oxford, Ohio, United States; The University of Texas Tyler, Tyler, Texas, United States; The University of Texas Tyler, Tyler, Texas, United States; The University of Texas Tyler, Tyler, Texas, United States; The University of Texas Tyler, Tyler, Texas, United States; The University of Texas Tyler, Tyler, Texas, United States

## Abstract

VESTA (Virtual Economic Simulation and Trust Assessment) is a groundbreaking assessment tool designed to address the escalating challenges faced by older adults in the realm of modern financial capacity and digital literacy. VESTA replicates real-world scenarios in a smartphone setting to assess older adults’ ability to safely navigate virtual financial transactions, detect financial scams, and share information securely. This preliminary study explores older adults’ (N = 20) initial reactions and 5-point ratings of VESTA. Results indicate that older adults positively rated VESTA as applicable to real life (M = 3.70, SD = 0.92) with realistic representations of messaging, mobile banking, and a smartphone layout – demonstrating the effectiveness of VESTA in simulating real-world scenarios. Older adults agreed that the application provides a good assessment of users’ risk for financial scams (M = 4.05, SD = 0.76), as well as their money management, ability with banking technology, trust judgment, and ability to share information safely. Additionally, older adults viewed VESTA as easy to use, easy to interact with, easy to understand, and an enjoyable gameplay experience, reaffirming its applicability across diverse older adult populations. Older adults positively rated VESTA as useful for everyday life by gathering useful information, testing abilities with financial technology, and educating people on their risk for financial scams (M = 4.70, SD = 0.57). In the modern financial landscape dominated by new technologies, VESTA enhances financial literacy and detects scam susceptibility among older adults, offering tangible benefits for their financial well-being in the modern financial technology landscape.

